# Microglial Activation Is Associated With Vasoprotection in a Rat Model of Inflammatory Retinal Vasoregression

**DOI:** 10.3389/fphys.2021.660164

**Published:** 2021-04-26

**Authors:** Sarah Riemann, Matthias Kolibabka, Stephanie Busch, Jihong Lin, Sigrid Hoffmann, Norbert Gretz, Yuxi Feng, Paulus Wohlfart, Hans-Peter Hammes

**Affiliations:** ^1^5th Medical Department, Medical Faculty Mannheim, Heidelberg University, Mannheim, Germany; ^2^European Center for Angioscience (ECAS), Medical Faculty Mannheim, Heidelberg University, Mannheim, Germany; ^3^Medical Research Center, Medical Faculty Mannheim, Heidelberg University, Mannheim, Germany; ^4^Experimental Pharmacology, European Center for Angioscience, Medical Faculty Mannheim, Heidelberg University, Mannheim, Germany; ^5^Sanofi Aventis Deutschland GmbH, TA Diabetes R&D, Frankfurt, Germany

**Keywords:** retinopathy, pericyte, microglia, vasoregression, clodronate

## Abstract

Vascular dysfunction and vasoregression are hallmarks of a variety of inflammatory central nervous system disorders and inflammation-related retinal diseases like diabetic retinopathy. Activation of microglia and the humoral innate immune system are contributing factors. Anti-inflammatory approaches have been proposed as therapies for neurovascular diseases, which include the modulation of microglial activation. The present study aimed at investigating the effects of microglial activation by clodronate-coated liposomes on vasoregression in a model of retinal degeneration. Clodronate treatment over 5 weeks led to an increase in activated CD74^+^ microglia and completely prevented acellular capillaries and pericyte loss. Gene expression analyses indicated that vasoprotection was due to the induction of vasoprotective factors such as *Egr1*, *Stat3*, and *Ahr* while expression of pro-inflammatory genes remained unchanged. We concluded that activated microglia led to a shift toward induction of pleiotropic protective pathways supporting vasoprotection in neurovascular retinal diseases.

## Introduction

The evolution of neurodegeneration and vascular dysfunction is a hallmark of a variety of central nervous disorders including Alzheimer’s disease, stroke, and retinal diseases like diabetic retinopathy (Marques et al., 2013; Prakash et al., 2013; Hammes, 2018; Jiang et al., 2018). In Alzheimer’s disease, microglia play a major pathogenic role (Hong et al., 2016; Molteni and Rossetti, 2017). It is proposed that microglia, activated by degenerating neurons, communicate inflammation into the neurovascular unit and ultimately cause vascular dysfunction and vasoregression (Kisler et al., 2017a). Similar mechanisms are involved in the pathogenesis of inflammatory retinal diseases such as diabetic retinopathy (Duh et al., 2017; Xu and Chen, 2017). As the retina and the brain are similar in the structure of the neurovascular unit, the eye can be used as a biomarker for pathogenic processes in the central nervous system (Lim et al., 2016). Preclinical and clinical studies show vascular alterations such as vascular dysfunction and vasoregression in both diabetic retinopathy and Alzheimer’s disease (Hammes et al., 2011; Kisler et al., 2017b; Jiang et al., 2018). The rat model of polycystic kidney disease (PKD) mimics several important changes in the neurovascular unit as observed in both diseases (Feng et al., 2009; Busch et al., 2012). Retinal neurodegeneration starting in the photoreceptor layer, accompanied by glial activation and increased expression of pro-inflammatory cytokines, results in pericyte loss and formation of acellular capillaries (Gallagher et al., 2006; Feng et al., 2009). It is highly likely that the pro-inflammatory switch of activated microglia induced by degenerating neurons causes, at least in part, vascular damage as observed in the PKD rat (Feng et al., 2011; Zhao et al., 2018). In this model, activated microglia near acellular capillaries showed an increased expression of the MHC-II invariant chain CD74, which was also observed in other models for diabetic retinopathy accompanied by similar neurovascular phenotypes (Feng et al., 2011; Wang et al., 2014; Schlotterer et al., 2018).

Clodronate is a bisphosphonate approved for the reduction of vertebral fractures and fracture-related pain in osteoporosis. In experimental models, liposomal clodronate is used to selectively deplete macrophages (Van Rooijen and Sanders, 1994; Moreno, 2018). Numerous studies have also shown not only that the depletory effect is only transient and that the respective tissue is repopulated with microglia over the course of 7–15 days but also that clodronate liposomes activate microglia, too (Checchin et al., 2006; Zelinka et al., 2012; Bollaerts et al., 2019; Hanlon et al., 2019). Therefore, we analyzed the effects of a microglia-modulating therapy on vasoregression in PKD rats using clodronate-coated liposomes. With this approach, we shed light on the contribution of microglia to vasoregression in the neurovascular unit.

## Materials and Methods

### Animals and Intravitreal Injections

Male homozygous transgenic PKD rats overexpressing a truncated polycystin-2, TGR(CMV-PKD2[1/703]HA), were used in this study (Gallagher et al., 2006). The animals were kept in a 12 h light–dark cycle with food and water *ad libitum*. At 4 and 8 weeks of age, animals were intravitreally injected with either 3 μl clodronate-coated liposomes or control liposomes (both from Encapsome, Brentwood, Tennessee, United States) under isoflurane anesthesia. At 9 weeks of age, 1 week after the second injection, animals were sacrificed, and the eyes were enucleated, snap-frozen in liquid nitrogen, and stored at –80°C. Uninjected, age-matched PKD and Sprague Dawley (SD) rats served as controls (Janvier Labs, Le Genest-Saint-Isle, France). All animal experiments were conducted in adherence to the EC directive 2010/63/EU and the guidelines of the statement for the use of animals in ophthalmic and visual research (ARVO) and have been reported following the ARRIVE guidelines. This study was approved by the federal animal welfare committee (Regierungspräsidium Karlsruhe, Karlsruhe, Germany).

### BV2 Cell Culture and MTT Assay

For the *in vitro* experiment, mouse brain microglial cells, immortalized by transfection with a v-raf/v-myc carrying J2 retrovirus (BV2 cells), were used (kindly provided by Prof. Ralph Lucius, University of Kiel). Cells were cultured in Dulbecco’s modified eagle medium (DMEM, Thermo Fisher Scientific, Darmstadt, Germany) containing 10% fetal bovine serum (Thermo Fisher Scientific, Darmstadt, Germany) and 1% penicillin/streptomycin (Thermo Fisher Scientific, Darmstadt, Germany). Confluent cells were detached using trypsin (Thermo Fisher Scientific, Darmstadt, Germany), seeded on a 96-well plate at 10^4^ cells per well, and grown for 24 h. Then, the cells were stimulated with different concentrations of either clodronate-coated or control liposomes ranging from 0.5 to 500 μM for 24 h. The medium was changed, 3-(4,5-dimethyl-2-thiazolyl)-2,5-diphenyl-2*H*-tetrazolium bromide (MTT) (Sigma Aldrich, Munich, Germany) was added, and cells were incubated 4 h before they were lysed with lysis buffer (containing 10% SDS and 0.02 M HCl) overnight. Absorbance at 550 nm was measured with a microplate reader (Tecan, Männedorf, Switzerland).

### Microglia Quantification

Microglial numbers and the fraction of activated microglia were quantified by immunofluorescence staining of retinal whole-mount preparations. Eyes were fixed in 4% formalin for 24 h, and retinae were isolated. After being washed thrice in phosphate-buffered saline (PBS), retinae were blocked and permeabilized in PBS containing 0.5% Triton X-100 and 1% bovine serum albumin for 30 min. Afterward, retinae were sequentially incubated with primary antibodies for *Iba*1 (Wako Chemicals, Japan) and CD74 (Santa Cruz, United States), as well as lectin for vascular staining (from *Bandeiraea simplicifolia*, Sigma Aldrich, Germany). After each primary antibody, incubation overnight, and three times washing in PBS, retinae were incubated with the corresponding secondary antibodies donkey anti rabbit Alexa Fluor 555 (Life Technologies, United States), chicken anti goat Alexa Fluor 488 (Life Technologies, United States), and streptavidin Alexa Fluor 633 (Life Technologies, United States) for 1 h. After the last washing step, the retinae were incised four times and flat mounted with the Vectashield Hard Set Mounting Medium (Vector Labs, United States). Images were obtained in five randomly selected fields in all three capillary layers with a confocal microscope (Leica TCS SP8, Wetzlar, Germany). Iba1^+^ cell somata were counted as microglia with lectin positivity as quality control. CD74^+^ cell somata or processes traceable to an Iba1^+^ cell soma were counted as CD74^+^ microglia. The microglial numbers of all three layers were summed up for each field and calculated as microglia per square meter of retinal area.

### Quantitative Retinal Morphometry

Quantitative retinal morphometry was performed on retinal digest preparations to evaluate numbers of acellular capillaries (AC/mm^2^ retinal area) and pericytes (PC/mm^2^ capillary area), according to a previously published protocol (Dietrich and Hammes, 2012). In short, eyes were enucleated and fixed in 4% formalin for 48 h. Retinae were isolated and washed in distilled water for 24 h. Then retinae were digested in 2.5% trypsin at 37°C for 2–4 h, until the outer retinal cells could be easily rinsed away with a water jet from a syringe. The retinae were transferred to slides, and the cells were rinsed away as described until only the retinal capillary layers were left. The preparations were then air-dried and PAS stained. For the quantification, images were obtained at 400× magnification in 10 randomly selected fields within the middle third of the retina. Acellular capillaries were counted and calculated as acellular capillaries per square meter of retinal area. Pericytes were identified by the darker nuclei in typical position, straight capillary pericytes on top of endothelial layers, and branch point pericytes in capillary branches. Pericytes were quantified as pericytes per square meter of capillary area, as calculated by the software used for quantification (CellF, Olympus, Hamburg, Germany).

### Microfluidic Card-Based Gene Expression Analysis

RNA was isolated from the retinae using a TRIzol reagent according to the manufacturer’s protocol (Invitrogen, Carlsbad, CA, United States). The quantity and quality of the isolated RNA were controlled using an RNA 6000 nanokit (Agilent, Waldbronn, Germany). Only samples with an RNA integrity number (RIN) > 7.5 were used for further analysis. Reverse transcription was performed using a high-capacity RNA-to-cDNA kit (Applied Biosystems, Weiterstadt, Germany). Then a quantitative pre-amplification step of 14 cycles followed, using a TaqMan Preamp Master Mix Kit (Applied Biosystems, Weiterstadt, Germany). Finally, real-time PCR of 48 genes was performed in parallel using TaqMan microfluidic card technology in a ViiA 7 thermocycler (Thermo Fisher Scientific, Darmstadt, Germany) with a maximum of 40 cycles. These cards contained wells for 44 specific genes and four reference genes (Supplementary Table 1). All hydrolysis probes were tested before by dilution experiments and used only when amplification efficacies were close to 100%. Threshold quantification cycles (Cq values) were obtained for each gene delivered by the manufacturer’s ViiA 7 software and further analyzed using the Array Studio software package (Version 9, OmicSoft Corporation, Research Triangle Park, NC, United States). The levels of gene expression were obtained by subtracting from the individual Cq values an average normalizing value of four reference genes. All data are available under the GSE165743 in the GEO.

### Quantitative Single-Gene RT-PCR

Retinal RNA was isolated and homogenized in a TRIzol reagent (Invitrogen, Carlsbad, CA, United States). RNA was reversely transcribed with the QuantiTect Reverse Transcription kit (Qiagen GmbH, Hilden, Germany) and subjected to qPCR analysis using hydrolysis probes (TaqMan probes, Applied Biosystems, Weiterstadt, Germany). Gene expression was analyzed by the comparative ΔΔCq method using *Actb* and *Gapdh* as reference genes (Schmittgen and Livak, 2008). All primers and probes were purchased from Applied Biosystems (Thermo Fisher Scientific, Weiterstadt, Germany, Supplementary Table 2).

### Western Blots

Retinae were isolated and homogenized in 0.1% SDS lysis buffer, and protein concentration was determined by Bradford assay (Bio-Rad Laboratories, München, Germany). Proteins were subsequently separated in a 4–20% gradient TGX Gel and immunoblotted to a polyvinylidene difluoride membrane using a semi-dry turboblot system (all from Bio-Rad, München, Germany). After blocking unspecific binding using 5% nonfat dry milk in TBS, containing 0.1% Tween (Sigma-Aldrich, Darmstadt, Germany), the membranes were sequentially incubated with the primary antibodies against pErk1/2, Erk1/2, pAkt, and Akt (all from Cell Signaling Technology, Frankfurt/Main, Germany) at 4°C overnight. After the washing steps, a horseradish peroxidase-conjugated swine anti-rabbit antibody was used for immunodetection (DakoCytomation, Hamburg, Germany). Immunoreactive bands were visualized by incubation in a chemiluminescence reagent (PerkinElmer, Boston, MA, United States), and signals were detected with the Fusion SL (VWR, Darmstadt, Germany). Membranes were stripped with a reprobing buffer containing 20% sodium dodecyl sulfate between immunodetection and incubation with subsequent primary antibodies. Integrated densities were measured with the ImageJ software (Abràmoff et al., 2004).

### *In silico* Analyses

*In silico* upstream effector analyses were performed using Enrichr (Chen et al., 2013; Kuleshov et al., 2016). For transcription factors, the ChEA 2016 and ENCODE TF ChIP-Seq 2015 databases were used (ENCODE Project Consortium, 2004; Lachmann et al., 2010). Transcription factors with *p* < 0.05 were selected. Regulatory gene network analyses were performed using GNCPro^TM^ (Gene Network Central, SABiosciences, Frederick, MD, United States^1^).

### Experimental Design and Statistical Analysis

Experimental group sizes were calculated following an established procedure according to the “Guidelines for the Care and Use of Mammals in Neuroscience and Behavioral Research” as previously described (Dell et al., 2002).

Data are presented as mean ± standard deviation. Differences between groups were analyzed with one-way ANOVAs with Tukey’s *t*-test to adjust for multiple comparisons. For the microfluidic card-based gene expression analysis, a one-way ANOVA with subsequent Benjamini–Hochberg false discovery rate (FDR) correction was performed. Statistical analysis was performed using GraphPad Prism (GraphPad Software v7.01, San Diego, CA, United States). A *p*/FDR < 0.05 was considered statistically significant.

## Results

### Intravitreal Clodronate-Coated Liposome Injections Induce Microglial Activation *in vivo*

**FIGURE 1:**
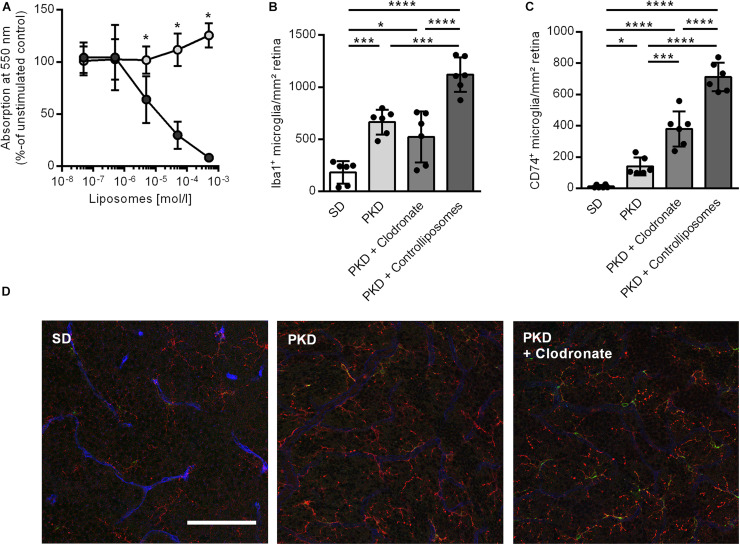
Effects of liposomes on microglia *in vitro* and *in vivo*. **(A)** Clodronate liposomes (dark gray) lead to a concentration-dependent reduction in cell viability in BV2 microglia *in vitro* compared to control liposomes (light gray) in an MTT assay. *p < 0.05. **(B–D)** Clodronate liposome injection showed no effect on the total number of retinal microglia as assessed by Iba1 staining **(B)** but led to an increase in activated CD74^+^ microglia **(C)**. Injection of control liposomes increased both total **(B)** and CD74^+^ microglia **(C)** compared to controls as well as clodronate-treated animals. Representative images of the deep capillary network **(D)** in SD, PKD, and PKD + clodronate rats show the increase in Iba1^+^ (red) microglia in both PKD and PKD + clodronate rats, whereas the number of CD74^+^ (green) microglia is further increased in PKD + clodronate rats compared to PKD rats. Blue = lectin for endothelial staining. *p < 0.05, ***p < 0.001, ****p < 0.0001. Scale = 100 μm.

**FIGURE 2:**
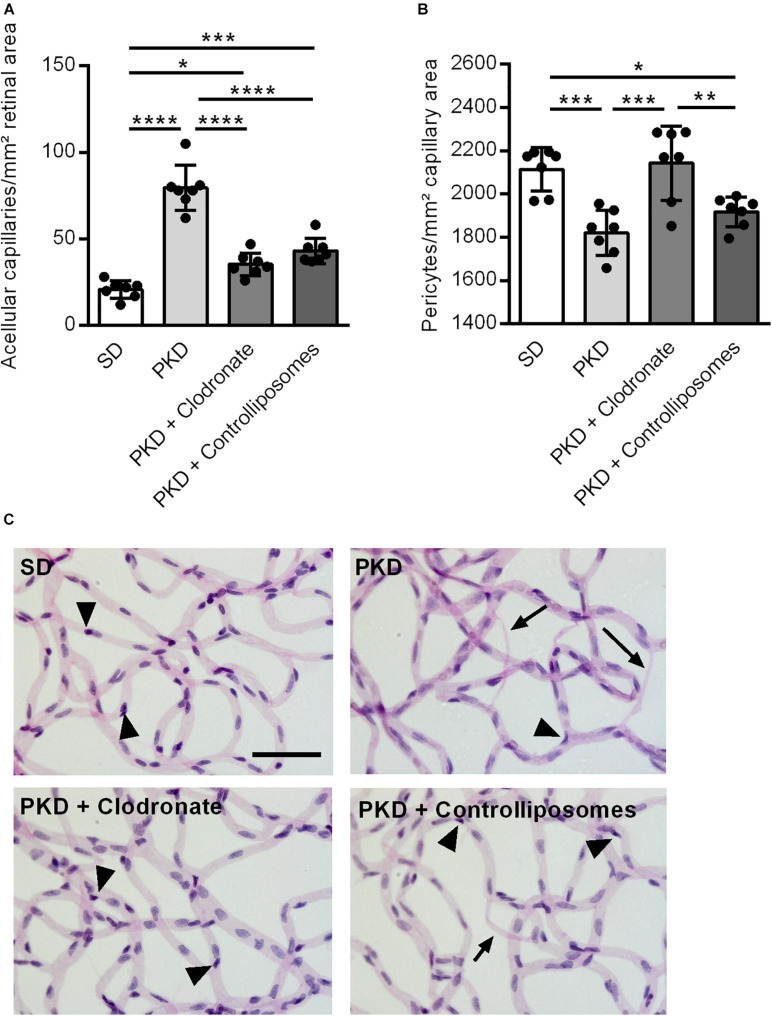
Vasoprotection in clodronate-treated PKD rats. **(A)** The number of acellular capillaries was significantly increased in PKD compared to SD rats. Upon liposome injection, the formation of acellular capillaries was almost completely prevented. **(B)** In PKD rats, the number of pericytes was significantly reduced compared to that in SD rats. This was completely prevented in clodronate-treated PKD rats, whereas control liposomes showed no effects on pericyte loss. **(C)** Representative retinal digest preparations showing acellular capillaries in PKD, PKD + clodronate and PKD + control liposome rats. *p < 0.05, **p < 0.01, ***p < 0.001, ****p < 0.0001. Arrow = acellular capillary, arrowhead = pericyte. Scale = 50 μm.

We first analyzed the effect of clodronate-coated liposomes on microglial viability *in vitro*, using BV2 microglial cells in an MTT assay. From a concentration of 5 × 10^–6^ mol/L upward, clodronate-coated liposomes significantly decreased the viability of the BV2 cells (Figure 1A). However, after two intravitreal injections into PKD rats, clodronate-coated liposomes showed no effect on total microglial numbers (Figure 1B). Instead, the numbers of activated CD74^+^ microglia were increased (Figure 1C). Injection with control liposomes significantly increased the number of both total and CD74^+^ microglia compared to that in uninjected and clodronate-coated liposome-injected animals (Figures 1B–D). Unexpectedly, the increase in activated microglia was accompanied by vasoprotective effects. Injection with control liposomes reduced the number of acellular capillaries to normal levels, independent of clodronate (Figures 2A,C). However, pericyte loss was prevented only in clodronate-coated liposome-injected PKD rats (Figure 2B,C).

### Gene Expression Analysis Identified *Fgf2* Downregulation as a Possible Effector System

**FIGURE 3:**
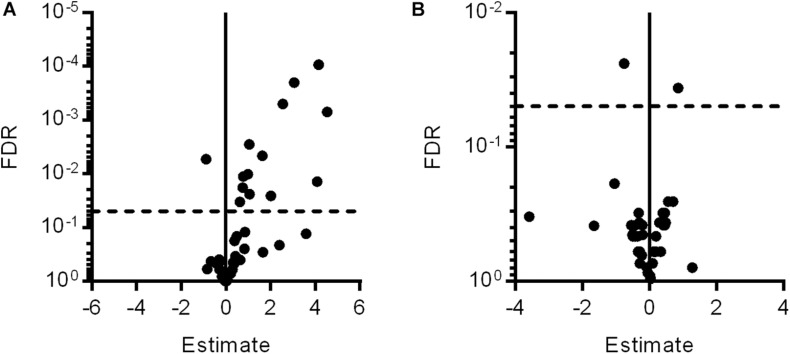
Gene expression changes after clodronate treatment in PKD rats. With microfluidic cards, the expression levels of 44 selected genes associated with retinal inflammation and vasoregression were analyzed. **(A)** Of the 44 specific genes measured, 13 genes were upregulated and one gene was downregulated in PKD rats compared to SD rats. **(B)** After clodronate injection, C1s was upregulated and Fgf2 downregulated compared to PKD rats, with only Fgf2 being regulated in PKD compared to SD rats. Horizontal dashed lines at FDR = 0.05 indicate the level of significance.

To identify factors involved in the mechanistic coupling of microglial activation and pericyte protection, an expression analysis for 44 genes associated with retinal inflammation and vasoregression was performed. Fourteen genes were significantly regulated in PKD rats compared to SD rats, which were predominantly innate immune system and interleukin-6 signaling-associated factors (Figure 3A and Table 1). Upon treatment with clodronate-coated liposomes, two genes were expressed significantly differently compared to untreated PKD rats, *C1s* (1.82-fold of PKD) and *Fgf2* (-1.68-fold of PKD; Figure 3B and Table 1). Of the 14 differently expressed genes comparing PKD and SD rats, only *Fgf2* showed a significant regulation upon clodronate treatment (8.32-fold of SD, Table 1). Treatment with control liposomes led to an increased expression of *C5ar2* and *Itgam* compared to clodronate-coated liposomes, but neither of these two genes was expressed differently between clodronate-coated liposome-treated and untreated PKD rats (Supplementary Table 1 and Table 1). To analyze whether the partial reversion of *Fgf2* upregulation in PKD rats upon treatment with clodronate-coated liposomes resulted in significant downstream regulation associated with vasoprotection, we assessed the activity of *Fgf2*-related signaling.

**TABLE 1:** Differentially expressed genes associated with inflammation and vasoregression.

**Gene**	**PKD vs. SD fold change**	**PKD vs. SD FDR**	**PKD + Clodronate vs. PKD fold change**	**PKD+ Clodronate vs. PKD FDR**
*Edn2*	23.13	0.00070	*−1.36*	*0.38490*
*Socs3*	17.75	0.00009	*1.37*	*0.36140*
*Serpina3n*	16.92	0.01390	*−1.21*	*0.73220*
*Fgf2*	8.32	0.00020	−1.68	0.02390
*Gfap*	5.85	0.00050	*1.10*	*0.60120*
*Lcn2*	4.04	0.02550	*−1.04*	*0.86220*
*Chi3l1*	3.11	0.00460	*−1.28*	*0.46100*
*C3*	2.09	0.02390	*1.38*	*0.30940*
*Tnfrsr12a*	2.07	0.00280	*1.24*	*0.36580*
*Icam1*	1.98	0.01010	*1.07*	*0.73220*
*Tyrobp*	1.71	0.01120	*1.01*	*0.90860*
*Itgam*	1.69	0.01800	*−1.15*	*0.38140*
*Cntf*	1.55	0.03320	*−1.05*	*0.78840*
*C1s*	*1.29*	*0.46320*	1.82	0.03640
*Bdnf*	−1.85	0.00530	*−1.42*	*0.44990*

*Genes are ranked based on the fold change between PKD and SD rats. Only genes with an FDR < 0.05 in either comparison are shown. Numbers in italic indicate non-significant regulation. FDR, Benjamini–Hochberg false discovery rate; SD, Sprague Dawley; PKD, polycystic kidney disease rat.*

### Modulation of *Mapk1* and Akt Signaling Pathways in Clodronate-Treated PKD Rat Retinae

**FIGURE 4:**
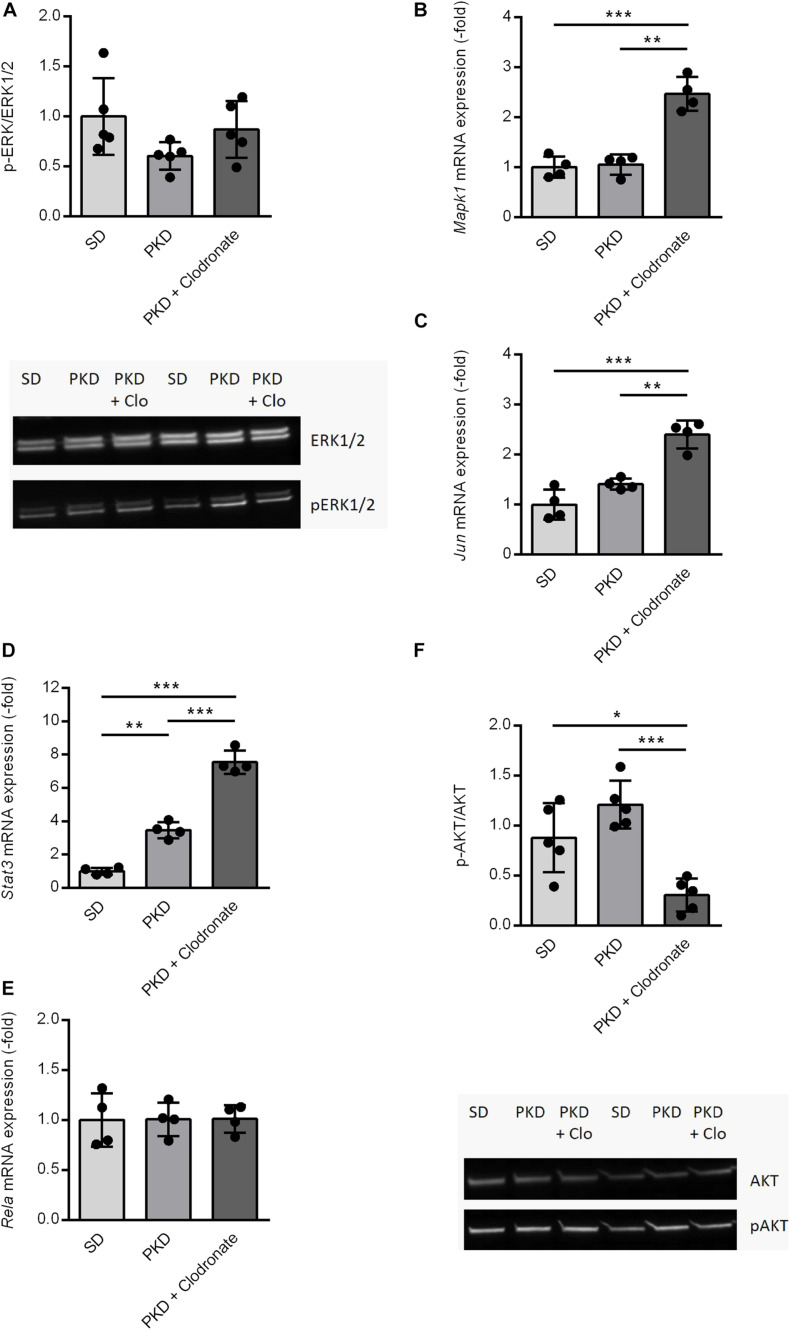
Expression and activation changes of Fgf2-related factors and pathways. **(A)** The activation of the MAPK/ERK1/2 pathway showed no significant differences between SD, PKD, and PKD+ clodronate rats as assessed by western blots for ERK1/2 and phosphorylated (p)ERK1/2. **(B–E)** Clodronate treatment led to an induction of Mapk1 **(B)** and Jun **(C)** expressions and increased the expression of Stat3 **(D)** compared to PKD rats, whereas the mRNA expression of Rela **(E)** showed no differences between the three groups. **(F)** The activity of the PI3K/AKT signaling pathway was significantly reduced in clodronate-treated PKD rats compared to both SD and PKD rats, indicated by the decreased phosphorylated (p)AKT/AKT ratios in the western blot analysis. *p < 0.05, **p < 0.01, ***p < 0.001. PKD + Clo = PKD + Clodronate.

Because the activation of the MAPK/ERK signaling pathway is a crucial factor mediating FGF2-related signaling, the phosphorylation of ERK was analyzed (Besser et al., 1995). In PKD rats, no differences in the activation of ERK signaling were observed compared to SD rats. Clodronate treatment showed no effects on the phosphorylation of ERK in PKD rats (Figure 4A).

On the transcriptional level, no differences in *Mapk1* expression were observable between SD and PKD rats, but in clodronate-treated PKD rats, the expression of *Mapk1* was significantly increased (Figure 4B). Independent of its kinase activity, *Mapk1* can inhibit the expression of IFNG-induced genes, but the expression of *Icam1*, which was the only IFNG-dependent gene that increased in PKD rats, was not affected by clodronate treatment (Table 1; Hu et al., 2009). As demonstrated here, the most abundant signal translation of FGF2 was not altered in clodronate-treated animals. Therefore, a gene network analysis was conducted in which the gene regulation observed in the microfluidic cards and the *Mapk1* expression were analyzed.

Associated with the observed changes in gene expression, inflammation, and neurovascular protection, *Jun*, *Stat3*, and *Rela* were identified as possible regulators.

The neuroprotective factor *Jun* was not differently expressed in PKD compared to SD rats. However, in clodronate-treated PKD rats, the expression of *Jun* was significantly increased (Figure 4C). *Stat3*, a co-inducer of microglial activation and transcriptional regulator of pro- and anti-inflammatory signaling factors, was significantly upregulated in PKD compared to SD rats, which was further increased upon clodronate treatment (Figure 4D). *Rela* showed no differences in mRNA expression between the three groups (Figure 4E). With the observed regulations, the question of AKT activation arose, as AKT is a crucial signaling pathway involved in pro- and anti-inflammatory signaling as well as in the regulation of the expression of *Mapk1* and *Jun* dependent on *Stat3* expression. Between SD and PKD rats, no differences in AKT phosphorylation were observed. However, clodronate treatment resulted in a significant reduction of AKT phosphorylation compared to untreated PKD rats, indicating decreased PI3K/AKT activity (Figure 4F). This change in PI3K/AKT activity in clodronate-treated animals indicates that the changes in previously unaltered signaling pathways might be responsible for the observed vasoprotection rather than a simple reversion of pathway activity changes.

### Upregulation of *Ahr*-Interacting Protective Factors

**FIGURE 5:**
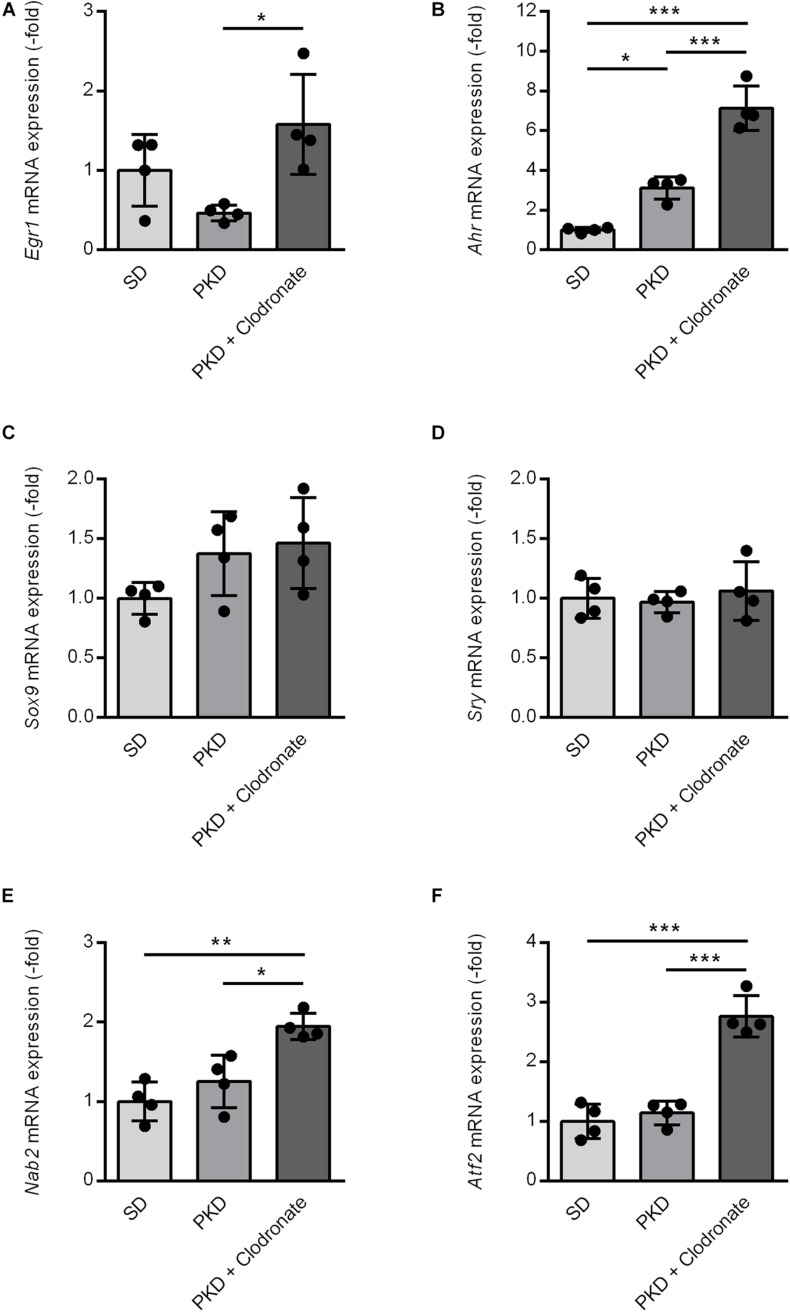
Induction of vasoprotective and neuroprotective factors in clodronate-treated PKD rats. The mRNA expression of Egr1 **(A)**, Nab2 **(E)**, and Atf2 **(F)** was significantly increased compared to SD and PKD rats. The upregulation of Ahr **(B)** in PKD compared to SD rats was further enhanced in clodronate-treated animals. Sox9 **(C)** and Sry **(D)** showed no differences in mRNA expression between the three groups. *p < 0.05, **p < 0.01, ***p < 0.001.

Upstream effector analysis of the regulation observed in the microfluidic cards and the qPCRs identified six transcription factors, *Egr1*, *Ahr*, *Sox9*, *Sry*, *Nab2*, and *Atf2*, interacting with *Stat3* and *Jun*, which are associated with cell protection and inflammation. For verification of the prediction, qPCRs were performed. Clodronate injection resulted in an increased *Egr1* expression compared to PKD controls, but not compared to SD rats (Figure 5A). Expression of the inflammation and microglial-activation-associated *Ahr* was significantly higher in PKD than in SD rats. Clodronate treatment resulted in an additional increase in *Ahr* mRNA expression in PKD rats (Figure 5B). The *Stat3*-inducing transcription factors *Sox9* and *Sry* showed no differences in regulation between the three groups (Figures 5C,D). The *Egr1*-induced *Egr1* repressor *Nab2* was significantly upregulated in clodronate-injected PKD rats (Figure 5E). *Atf2*, which itself has neuroprotective effects and is induced by *Ahr*, while regulating *Stat3* and *Egr1* expressions, was significantly increased in PKD rats upon clodronate treatment (Figure 5F).

These results showed that *Ahr*-interacting neuroprotective and vasoprotective factors were either induced or further increased upon clodronate injection and microglial activation.

## Discussion

In this study, we demonstrate vasoprotective effects associated with clodronate-coated liposome-induced microglial activation. Vasoprotection was accompanied by the induction of *Ahr*-dependent protective factors in the course of microglial activation, which are possible effectors mediating the vasoprotective effects. The partial depletion of microglia in relation to liposome-dependent activation and proliferation of microglia is crucial in this process, as control liposomes decreased the formation of acellular capillaries but failed to protect the more important pericytes. The partial depletory effects are suggested by the increased numbers of Iba1^+^ and CD74^+^ microglia in PKD rats injected with control liposomes.

The pericyte protective effects were not mediated via regulation of classical inflammatory pathways, as only minimal changes were observed in the expression of the selected candidate genes. Instead, the gene expression analysis showed that the clodronate injection did not simply reverse the changes occurring in the PKD rat compared to SD rats but apparently activated alternative pathways which might be either modulating or modulated by the microglial subpopulations driving the inflammation (Cherry et al., 2014).

The activation of microglia is a common process in diabetic retinopathy and in models reflecting the various hallmarks of its pathogenesis (Zeng et al., 2008; Karlstetter et al., 2015; Stitt et al., 2016; Mazzeo et al., 2017). Activated microglia were identified to induce and mediate effects detrimental to the vasculature (Krady et al., 2005; Kezic et al., 2013; Portillo et al., 2014, 2017; Mazzeo et al., 2017). In contrast, in our study, the increase in microglial activation upon clodronate injection counterintuitively reduced vasoregression. In general, activated microglia can have both pro- and anti-inflammatory properties. Our gene expression analysis indicated that clodronate treatment did not simply result in an obvious shift in microglial polarization (Stefater et al., 2011; Cherry et al., 2014; Arroba et al., 2016). Instead, alternative inflammation-associated factors were induced, which probably are responsible for the observed vasoprotection. Whether these protective factors were induced in microglia directly or in other neurovascular compartments has to be addressed in subsequent studies. Furthermore, as the gene expression analysis was performed with whole retinal lysates and a predefined limited set of genes, microglial subpopulation shifts cannot be ruled out. Focused analyses, e.g., using single-cell RNA sequencing of isolated microglia, could provide a more detailed insight into the behavior of microglial subpopulations upon clodronate treatment.

In our view, the downregulation of *Fgf2* upon clodronate treatment is unrelated to the treatment effect, as its expressional change was small compared to the difference between untreated PKD and SD rats. In our study, the upregulation of *Mapk1* could be a result of the reduced *Fgf2* expression, as *Fgf2* can reduce the expression of *Mapk1* in a complex induction-repression dynamic via multiple negative feedback loops, but *Mapk1* expression showed no difference between SD and untreated PKD rats despite an eightfold increase in *Fgf2* expression (Smith et al., 2006; Lake et al., 2016). Moreover, as the activity of AKT was not increased but decreased in clodronate-treated PKD rats, the inducer of *Mapk1* expression remains to be determined.

As the activity of MAPK/ERK1/2 was not changed upon clodronate treatment, the vasoprotection could be related to the increased *Mapk1* expression independent of its kinase activity. *Mapk1* can act as a transcriptional repressor of IFNG-induced genes (Hu et al., 2009). However, the expression of the IFNG-induced *Icam1*, which was also upregulated in PKD compared to SD rats, was not affected by clodronate treatment, despite the increased *Mapk1* expression, which lets the repression of IFNG-induced genes by *Mapk1* seem unlikely in this context.

The *Mapk1* and AKT-dependent *Jun*, as well as the *Mapk1*-dependent AKT inducer *Stat3*, were both significantly increased in clodronate-treated animals. The protein encoded by *Jun*, jun proto-oncogene, a transcription factor involved in JNK-mediated transcriptional changes, has been identified as a crucial part in the mediation of TLR4-dependent neuroinflammation and in the ocular degeneration occurring in an Alzheimer’s disease mouse model (Badshah et al., 2016; Buccarello et al., 2017). The expression of *Jun*, however, together with *Atf2*, which we identified as an additional factor induced by clodronate treatment, was found in retinal ganglion cells and can be neuroprotective (Kreutz et al., 1999). Additionally, the c-Jun pathway was identified as an antiapoptotic pathway in cerebral microvascular smooth muscle cells, which have similar properties as retinal pericytes (Qu et al., 2017). *Stat3*, which is induced in activated microglia, is another ambiguous transcription factor we identified as upregulated upon clodronate injections. The inhibition of *Stat3* and STAT3 signaling can reduce apoptosis of retinal endothelial cells and delays the vision loss observed in a rat model of diabetic retinopathy (Ye and Steinle, 2016; Vanlandingham et al., 2017). In contrast, *Stat3* is the mediator of the protective effects of TLR3 signaling under oxidative stress, the reduction of *Stat3* signaling contributes to mitochondrial dysfunction in endothelial cells, and *Stat3* in Müller cells is essential for retinal regeneration (Nelson et al., 2012; Patel and Hackam, 2014; Banerjee et al., 2017). In combination, in clodronate-treated PKD rats, the net effect of the induction of these ambiguously acting transcription factors is vasoprotection rather than detrimental, but the exact interaction of these factors has to be analyzed in subsequent studies, targeting the different components selectively.

The transcriptional activator *Egr1* was upregulated in clodronate-treated rats. The induction of *Egr1* leads to microglial activation and can contribute to endothelial dysfunction (Langmann et al., 2009; Karthikkeyan et al., 2018). In opposition, *Egr1* is essential in regulating apoptosis and was demonstrated to mediate neuroprotective effects (Stuart et al., 2005; Bakalash et al., 2011). The aryl hydrocarbon receptor (*Ahr*), which was highly upregulated in clodronate-treated PKD rats, can mediate both pro- and anti-inflammatory signals in activated microglia (Lee et al., 2015). Although *Ahr* was observed to be involved in blood–brain barrier alterations, the deletion of *Ahr* leads to increased microglial accumulations and atrophy of the retinal pigment epithelium (Chang et al., 2012; Kim et al., 2014).

The factors we found upregulated in clodronate-treated PKD rats have dual properties on the retina. The observed vasoprotection shows that the protective effects of these pathways clearly exceeded potential detrimental effects, while it remains unclear which of the pathways played the most dominant role. Endothelial and glial cells have been shown to influence microglia, which then can modulate pericytes (Redzic et al., 2015; Li et al., 2020). It is therefore possible that a change in the local concentration of pericyte-modulating factors, not detectable by a global assay like our microfluidic card analysis, was responsible for the protective effect that was associated with the increase in activated microglia shown in this study.

Whether clodronate treatment has a protective effect on the neuroretinal function could not be determined sufficiently, due to cataract formation after intravitreal liposome injection (Sampat and Garg, 2010). The effects on the retinal vasculature suggest a protection of neurovascular function in clodronate-injected animals, but this has to be verified in future studies.

In contrast to observations in other microglial depletion studies using clodronate liposomes, our long-term experiment revealed that the stimulatory effect of the liposomes outweighed the depletory capacity, providing an additional stimulus for the remaining microglia (Zhou et al., 2015; Fouda et al., 2018; Xu et al., 2018). We speculate that this effect was due to the experimental period of 5 weeks and to the numbers of injections applied. Previous studies used single injection and systemic clodronate treatment in different settings and observations, which is a likely explanation for observed discrepancies (Zhou et al., 2015; Fouda et al., 2018; Xu et al., 2018). Even though the microglial population was not reduced compared to untreated controls but the amount of activated CD74^+^ microglia was increased, the treatment demonstrated vasoprotective effects.

In summary, we demonstrated that increased microglial activation is associated with vasoprotection probably by induction of various pleiotropic pathways. The vasoprotection and especially the prevention of pericyte loss are probably due to a combination of a variety of pathways with potentially ambiguous effects, with the interaction of these pathways resulting in a net protection of the retinal vasculature. The reduction in acellular capillaries in control liposome-injected animals shows that the liposomal activation alone is associated with partial vasoprotection. Whether the additional benefit of the partial microglial depletion in the clodronate-treated animals is due to an overall reduction in microglia, the depletion of a certain subtype, or a pleiotropic depletion-independent induction of vasoprotective factors in the neurovascular unit needs to be carefully addressed in future studies.

## Data Availability Statement

The datasets presented in this study can be found in online repositories. The names of the repository/repositories and accession number(s) can be found in the article/Supplementary Material.

## Ethics Statement

The animal study was reviewed and approved by the Regierungspräsidium Karlsruhe. Written informed consent was obtained from the owners for the participation of their animals in this study.

## Author Contributions

SR, MK, SB, and H-PH contributed to conception and design of the study. SR, MK, and PW contributed to the acquisition and analysis of the data. SR, MK, JL, YF, PW, and H-PH contributed to the analysis and interpretation of the data. SH and NG contributed to the interpretation of the data. SR and MK wrote the manuscript. All authors contributed to manuscript revision, read, and approved the submitted version.

## Conflict of Interest

PW was a full time employee of Sanofi Aventis Deutschland GmbH. Microfluidic cards based qPCR analyses were conducted at the research facility of Sanofi Aventis Deutschland GmbH. Experiments and communication was performed within the IRTG GRK 1874 DIAMICOM reviewed by the DFG. Sanofi Aventis had no influence on the interpretation nor the decision to publish these data. The remaining authors declare that the research was conducted in the absence of any commercial or financial relationships that could be construed as a potential conflict of interest.

## Footnotes

1 http://gncpro.sabiosciences.com/gncpro/gncpro.php
